# Answer to Scepticus

**Published:** 1804-02-01

**Authors:** 


*The following answer to Scepticus was receivd in the beginning of last May; but we then thought that such observations required no other answer than the very short one which appeared in the next Number. We now find however, that the same ideas are brought before the public with the pomp of a considerable quarto volume, written with great ingenuity and acuteness, and which excites very general attention. It is understood that elaborate answers to it are preparing; in the mean time we present our readers with the following short one to Scepticus.*

---

To the Editors of the Medical and Physical Journal.

Gentlemen,

YOUR Correspondent Scepticus (see vol. ix. p. 356) has given the world some ingenious remarks on the *danger* of exterminating the small-pox. As this idea has at least novelty to recommend it, let us examine his arguments in the order in which they stand.

It is very true that "though the faculty cannot boast the honour of introducing inoculation for the small-pox, they warmly interested themselves in it." If by *warmly*, your Correspondent means to refer to the regimen with which they connected the new practice, his expression is much too *cold*. For though the noble lady to whom we owe the

 discovery discovery, tells us that in Turkey the old women are the inoculators, and that *to take the small-pox* is, in that country, analogous to *taking the air*, yet no sooner did our physicians lay hold of the business than they shewed the *warmth* of their zeal by almost stifling their patients. Hence inoculation was in danger of being *stifled* in its birth; for though the practice was for the most part successful, yet the number of unsuccessful cases was sufficient to damp the courage of many very impartial thinkers.

At length the Suttons, under the pretence of a secret remedy said to be contained in their pills, introduced a more successful practice, which obliged all the regular faculty to look about them. At first they had recourse to the great danger which attends every bold innovation, and to discovering a few solitary instances in which the innovators had failed. But the public was not to be thus deceived, and the success of the Suttons was such as to secure them the reputation they deserved, till at last establishments were formed to which the young and old resorted, according to Lady W. Montague's expression, to take the air and the small-pox at the same time.

Matters now became serious. Surgeons kept their lancets only to bleed; physicians were no longer consulted for a complaint which had lost most of its terrors; and apothecaries neither sent in the preparative powders, the febrifuges nor opiates during the eruption and turn, nor the subsequent cathartics and sweeteners. Such a state of things could not long continue. The pills were analysed, and the cold regimen was discovered to be as old as Sydenham, though long since exploded by his *wiser* successors. Sutton therefore had no merit. His secret pills were no longer a secret, and his treatment was not his own discovery; still however the Suttons retained their character with the public, and their success entitled them to it, till by degrees their practice became universal, and every town, every village, had its stationary or its itinerant inoculator. At length an imperial physician and baron entered the lists, and the name of Sutton soon gave way to Dimsdale. It is but justice to add, that wherever Dimsdale is read, the name of Sutton will appear with not less respect than Æsop accompanies the elegant Phædrus.*

* See Present Method of Inoculating, &c. by T. Dimsdale, M.D. 1767, Introduction, p. 5. In a subsequent pamphlet, I believe, Account of Inoculation in Russia, the Suttons are mentioned by name as first introducing the cold system.

But

But though the success of the Suttons and the sanction of the faculty, who now adopted their practice, had robbed the disease of many of its terrors, still a few solitary failures were sufficient to deter parents from undertaking this task. Your correspondent therefore conceives, that the encouragement to vaccination "breathes a higher tone of disinterestedness," but still disposed to suspect all men of some view to their own interest, speaks of medical men puffing off the "safety of the cow-pox in the public prints, and themselves obliquely, as the only beings who know how to manage it."

If your Correspondent is ever so much disposed to humor, there are certain moments when all men think it right to be grave in the midst of all their raillery; I must therefore be allowed to remind him, that in the Inventor of Vaccination we see nothing but the most enlarged philanthropy, the highest degree of liberality, and that attention to the importance of his profession which suffers nothing to escape him. None of that fury of forcing the practice down the throats of others, nor a hint that it can be better performed by himself than his groom. He is therefore not answerable for the "intemperate zeal of others," or for the manner in which *one writer* suspects the integrity of all who venture to doubt what he himself is afraid of asserting; who renders himself ambiguous by first declaring that vaccination never produces secondary *pustules*, and then, in a round about way, admits that secondary *vesicles* sometimes appear. But enough of this. Let us endeavour to relieve Scepticus from his other apprehensions, without attending to those puffs which seem to offend him so much.

As he has not pointed out the precise passage in Mr. Malthus's work, he cannot expect me to pursue the ingenious enquiry relative to the geometrically progressive increase of population and the arithmetical ratio of increasing the productions of the earth; I shall therefore only remind him of the rapid increase in the population of America, yet that she is still in a condition to supply the West India Islands and a considerable part of Europe with food. I know it will be said that America is different from Europe, because new land may be continually broken up, and even the increase of population may be exaggerated, since Europe, whom she feeds, loses a part of her inhabitants to increase those of the new Continent, and perhaps, as the whites increase in number, the ancient Aborigines become extinct. For argument's sake therefore we will admit Mr. Malthus's propositions propositions and the inference of Scepticus, namely, that new diseases arise to thin the ranks of mankind and equalize the principles of generation and production;" still it will not be difficult to prove, that "a due equilibrium between these opposing forces may be effected in spite of the idle interference of meddling man," in preventing or lessening disease; and this without "the necessity of war, pestilence, or famine."

I need not a better authority than that philosopher whose words are so often quoted, which he put into the mouth of the *great* King of Brobdignag. "Show me the man who will produce two blades of corn where one used to grow, and to him I will erect statues." This writer lived in an Island whose population is large for its extent, whose climate is less genial than our own, whose inhabitants are pecularly prolific, yet whose soil produces food not only for its people, but for the navy of Great Britain and some of our foreign settlements. It appears however as if this philosopher had been fearful lest Ireland should be over populous even in his days. With a sagacity therefore worthy of the author, we find him not proposing to disable men by the great pox, or to destroy children by the small pox, (practices more brutal than Chinese mutilation) but to turn this increased population to a good account, and to make an increase of offspring a real blessing to the parents. Let every poor man, says he, sell his first child to be killed and served up at his landlord's, his landlord's steward's, or the steward of that steward's table. By this traffic and sale, he will gain money enough to feed well his wife whilst she is suckling her second child, so as to enable her to fatten it sufficiently for the great man's table, and so on till advance of years puts an end to the prolific powers of the mother and the riches of the father. Surely such a practice is kinder than to suffer the poor infants to languish with a loathsome disease which renders them more troublesome whilst living, and useless when dead. I am aware that one source of astonishment to your Correspondent still remains unexplained; "that the faculty should wish to lessen the danger and mortality of a disease." 'Tis evident by this stupid astonishment that he is not of the profession. I know not therefore whether it will be considered as justifiable to admit him into all our mysteries: of this, you Gentlemen Editors must judge. It does not indeed, require much ingenuity to see, that a patient, like a soldier, can only be useful whilst living. The facetious poet therefore advises the latter to run away, that he may live to fight again; again; and every prudent practitioner recollects that at the death of a patient he receives no thanks but from his heir, who, how well soever he may be satisfied, has not always the gratitude to employ the person to whom he is indebted for the early possession of the estate.

Perhaps Scepticus will ask, why then should I sanction the horrid practice of child murder, if we have an interest only in the living? Forgive me, good Messrs. Editors, if I betray our order a step further, to satisfy the kind anxieties of one who is so much alarmed, lest we should suffer by our great disinterestedness. I need not remind you that the slaughter I propose is not indiscriminate, like that of the cruel Herod, but confined to the children of the poor. These, we well know, are more plague than profit. Even our most miraculous cures among them rarely bring us into fashion; we may therefore gladly save ourselves all this trouble by turning them over to other practitioners, whose time is respected in courts of justice like our own. But lest I should say too much, let me stop abruptly.

Your's,

CREDULOUS.

